# Prognostic impact of lymph node characteristics after therapeutic neck dissection for classic N1 papillary thyroid cancer

**DOI:** 10.1093/bjsopen/zrad124

**Published:** 2023-11-28

**Authors:** Klaas Van Den Heede, Nele Brusselaers, Esmee Breddels, Sébastien Gaujoux, Camille Buffet, Fabrice Menegaux, Nathalie Chereau

**Affiliations:** Department of General and Endocrine Surgery, Pitié Salpêtrière Hospital, APHP, Sorbonne University, Paris, France; Department of General and Endocrine Surgery, Onze-Lieve-Vrouw (OLV) Ziekenhuis Aalst, Aalst, Belgium; Center for Translational Microbiome Research Department of Microbiology, Tumor and Cell Biology, Karolinska Institute, Stockholm, Sweden; Global Health Institute, University of Antwerp, Wilrijk, Belgium; Global Health Institute, University of Antwerp, Wilrijk, Belgium; Department of General and Endocrine Surgery, Pitié Salpêtrière Hospital, APHP, Sorbonne University, Paris, France; Groupe de Recherche Clinique no. 16 Thyroid Tumors, Sorbonne University, Paris, France; Groupe de Recherche Clinique no. 16 Thyroid Tumors, Sorbonne University, Paris, France; Thyroid and Endocrine Tumor Unit, Pitié Salpêtrière Hospital, APHP, Sorbonne University, Paris, France; Department of General and Endocrine Surgery, Pitié Salpêtrière Hospital, APHP, Sorbonne University, Paris, France; Groupe de Recherche Clinique no. 16 Thyroid Tumors, Sorbonne University, Paris, France; Department of General and Endocrine Surgery, Pitié Salpêtrière Hospital, APHP, Sorbonne University, Paris, France; Groupe de Recherche Clinique no. 16 Thyroid Tumors, Sorbonne University, Paris, France

## Abstract

**Background:**

The impact of lymph node characteristics on mortality and recurrence remains controversial. This study evaluated the prognostic impact of lymph node characteristics in a large, homogenous cohort of patients with therapeutic neck dissection for clinically N1 classic papillary thyroid cancer (PTC).

**Methods:**

All consecutive adult patients with therapeutic central and lateral neck dissection for PTC at a French referral centre were prospectively enrolled from January 2000 until June 2021. The primary outcome was the impact of lymph node characteristics in predicting a disease event (persistence or recurrence), using univariable and multivariable logistic regression modelling.

**Results:**

A total of 462 patients were included. Lymph node capsular rupture was seen in 260 patients (56.3 per cent). Median maximum lymph node size was 15 (i.q.r. 9–23) mm. The median central, lateral, and total lymph node ratio (LNR) was 0.50 (i.q.r. 0.22–0.75), 0.15 (i.q.r. 0.07–0.29), and 0.26 (i.q.r. 0.14–0.41), respectively. After a median follow-up of 93 (i.q.r. 50–149) months, 182 (39.4 per cent) patients had a disease event. After multivariable analysis, the number of harvested lymph node >35 (OR 2.33 (95 per cent c.i. 1.10–4.95)), presence of lymph node capsular rupture (OR 1.92 (1.17–3.14)), and total LNR >0.20 (OR 2.37 (1.08–5.19)) and >0.40 (OR 4.92 (1.61–15.03)) predicted a disease event. An LNR of 0.20 predicted a disease event with a sensitivity of 80.8 per cent and a specificity of 50.4 per cent.

**Conclusion:**

Disease persistence or recurrence after thyroidectomy with therapeutic neck dissection for classic PTC with preoperative nodal disease appears to depend on number of harvested lymph node, presence of lymph node capsular rupture, and total LNR.

## Teaser

This study aimed to evaluate the prognostic impact of lymph node (LN) characteristics in a large surgical cohort that underwent therapeutic neck dissection for N1 classic papillary thyroid cancer. Disease persistence or recurrence appears to depend on number of harvested LNs, presence of LN capsular rupture, and total LN ratio.

## Introduction

The prognosis of most patients with papillary thyroid cancer (PTC) is good, even for advanced stages, with a relative 10-year survival of 96 per cent and 90 per cent in women and men, respectively, and a 20-year overall survival of 90 per cent^1,2^. In a large American population less than 45 years old, the adjusted 20-year overall survival was 97 per cent even in the presence of nodal metastasis^3^.

Survival mainly depends on sex, age, presence of distant metastases, extrathyroidal extension, tumour size, clinical node metastasis, multifocality, and presence of nodal capsular rupture^2,4^. Given the favourable overall survival, disease recurrence and persistence might be more meaningful to clinicians and patients, as it potentially leads to intensified surveillance, administration of radioactive iodine (RAI) and/or additional surgery, and decreased quality of life^5^.

Predicting disease recurrence and the development of predictive tools have been challenging. The AJCC/UICC TNM (8th edition) staging system has been validated to predict overall survival and disease recurrence, but only includes the location of metastatic lymph nodes (LNs)^5,6^. The American Thyroid Association (ATA) risk stratification system is widely used and intended to predict recurrence, and includes risk factors such as the number and size of LN metastasis^7^. Additionally, the lymph node ratio (LNR), defined as the number of metastatic LNs to the total number of harvested LNs, has been shown to predict recurrence^8,9^, with higher ratios reflecting more aggressive malignant behaviour. Results of LNR on predicting disease-specific mortality and overall survival have been conflicting^10,11^. Previous studies evaluating LNR and disease recurrence exhibit small sample sizes and missing data, have mixed prophylactic and therapeutic neck dissections, do not evaluate the maximum LN size or the presence of nodal capsular rupture, and/or fail to analyse the central and lateral compartment separately^8,9,10,12^. Moreover, the definition of recurrence varies among different studies and often includes or excludes biochemical persistence, true persistence, or true recurrence^12,13^.

This study aimed to evaluate the prognostic impact of LN characteristics (central, lateral, and total LNR, maximum LN size, and presence of nodal capsular rupture) in a large, homogenous, surgical cohort of patients that underwent therapeutic central and lateral neck dissection for clinically N1 PTC in a tertiary referral centre.

## Methods

### Study cohort

All consecutive patients with classic PTC and preoperative LN involvement (N1) without distant metastases at diagnosis who underwent a total thyroidectomy with therapeutic central and lateral neck dissection and postoperative RAI (according to the current guidelines) were enrolled at a tertiary referral university hospital in France. Study enrolment lasted from January 2000 until June 2021 and required at least one year of follow-up. All patients provided preoperative written informed consent to participate in anonymous data collection for retrospective analyses. Patients with non-PTC and patients with a follicular, tall cell, hobnail, or diffuse sclerosing variant of PTC on final postoperative pathology were excluded from the analysis as they present different populations with different prognoses. In the current series, the impact of the Ki-67 index could not be evaluated. Moreover, no data on genetic mutational status were available. The surgical department is a high-volume centre specialized in endocrine surgery, with around 1400 thyroid surgical procedures every year. All cancer patients are discussed at a multidisciplinary team meeting. Data were retrieved from a prospectively maintained institutional database, approved by the ‘Comité de Protection des Personnes’ (an institutional review board). As this is a retrospective study with no new data collection, this study did not require ethical committee approval, in accordance with French recommendations for good clinical practice.

### Patient characteristics

Patient demographics (age at surgery, sex, height, weight, BMI, and family history of thyroid and other cancers (defined as the presence of one family member with (thyroid) cancer)) were collected. Preoperative thyroid-stimulating hormone (TSH, reference value 0.45–4.50 mIU/l), and the presence of hypo- or hyperthyroidism, arterial hypertension, hypercholesterolaemia, obstructive sleep apnoea syndrome, and diabetes were collected. Intake of vitamin K antagonists, aspirin, and steroids was registered.

### Preoperative investigations

All patients underwent preoperative neck ultrasound (US) evaluating thyroid nodularity, LN, and thyroid nodule size and appearance. US-guided fine-needle aspiration was performed to confirm the presence of PTC and/or LN metastasis. A patient was considered N1 when suspicious LNs were noted on clinical examination and/or imaging before surgery.

### Surgical technique and characteristics

All patients underwent a total thyroidectomy, with central (level VI) and lateral neck dissection (levels III, IV, and level II if the cancer was in the upper third of the thyroid lobe) on the side of the primary thyroid tumour and contralateral when there were biopsy-proven contralateral LNs. All patients were initially diagnosed as having PTC on preoperative US-guided fine-needle aspiration cytology or with a positive frozen section during surgery. A therapeutic contralateral lateral neck dissection was only performed in patients with proven or suspected LN metastasis. Time from referral to surgery (in days), date of surgery, identification of the RLN, and use of intraoperative nerve monitoring (IONM) were also collected.

### Pathology and clinical/surgical outcome data

Pathology reports included the main and secondary histopathological diagnosis, micro- or macrocarcinoma, multifocality, presence of bilateral thyroid disease, maximum tumour size (millimetres), tumour size of a second lesion, when present (millimetres), number of tumour foci, presence of thyroid capsular and vascular invasion, and presence of parathyroid glands, the weight of the resected specimen (grams), and the 8th edition of the AJCC/UICC TNM staging system. Registered LN characteristics included number of harvested LNs, number of positive LNs, LN capsular rupture, number of total and positive central and lateral LNs, and maximum LN size (millimetres). Total, central, and lateral LNR was defined as the number of positive LNs divided by the total number of LNs overall, or number in the central or lateral compartment, respectively.

Surgical and clinical outcome data included length of hospital stay, time to first and last surgical follow-up, mortality, recurrent laryngeal nerve (RLN) palsy (temporary/permanent), laryngoscopy results, hypoparathyroidism (temporary/permanent), oral calcium and/or vitamin D supplementation, re-intervention for bleeding, and wound infection. All procedures were performed under general anaesthesia. Postoperative laryngoscopy was performed selectively (on clinical grounds). RLN palsy and hypoparathyroidism were considered permanent when present 12 months after surgery.

### Study outcome

All patients were seen in person 6 weeks after surgery. All patients followed a standardized follow-up schedule that included physical examination, neck ultrasound, serum thyroglobulin (Tg), and Tg auto-antibody measurements after stimulation or under suppressive treatment at 6 and 12 months, and then annually thereafter. The primary outcome was the risk of a disease event, defined as: (a) a locoregional event (structural LNs in the central or lateral neck compartment or abnormal tissue in the thyroid bed cytologically confirmed by fine needle aspiration); (b) biochemical evidence of disease (stimulated Tg level > 10 ng/ml after thyroid hormone withdrawal or >5 ng/ml after recombinant human thyrotropin administration, progression of basal (suppressed) Tg and/or basal Tg > 1 ng/ml, using the same assay technique, appearance or progressive elevation (>50 per cent) of Tg auto-antibodies (TgAb), using the same assay technique, and/or isolated and repeatedly elevated serum Tg levels). Both persistent (within 12 months of surgery) and recurrent (event after complete remission) disease were considered a disease event.

### Statistics

Continuous variables are reported as medians and interquartile ranges, and nominal variables as counts and percentages. Descriptive statistics were used to compare differences between patients with and without a disease event after initial treatment, using the Chi-square test and the Mann–Whitney U test, as appropriate. Two-sided *P* < 0.05 indicates statistically significant differences. All continuous variables were categorized into three categories each including about one-third of the total cohort. Risk factors for a disease event were assessed using univariable and multivariable logistic regression modelling and presented as odds ratios and 95 per cent c.i. Variables in the multivariable logistic regression analysis were chosen when statistically significant in univariable analysis in addition to clinically relevant variables such as age and sex. As most disease events included biochemical and structural persistence, time-to-event analysis was not useful. Patients with missing values were excluded from the analysis, except for maximum lymph node size. To be able to perform a complete case analysis, a dummy value (missing data) was created. This value was not statistically significant in the univariable analysis. Univariable Cox proportional-hazards regression analysis was performed on the subgroup of true recurrence and presented as HR and 95 per cent c.i. Power was insufficient for multivariable regression on the subgroup of true recurrence. All statistical analyses were conducted using STATA® (StataCorp, V.16.1/MP).

## Results

### Patient and tumour characteristics

**Fig. 1:**
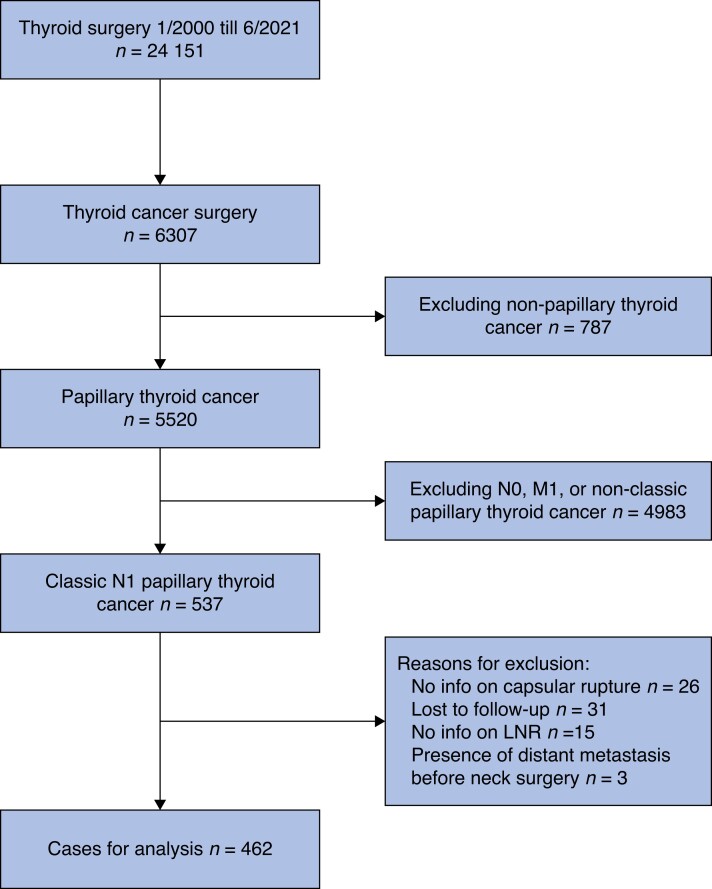
Flowchart

Throughout the study period, 24 151 thyroid surgical procedures were performed, of which 6307 procedures were for thyroid cancer. A total thyroidectomy with therapeutic central and lateral neck dissection was performed in 537 consecutive patients. Of these, 462 fulfilled all inclusion criteria. The reasons for exclusion are summarized in *Fig. 1*. The 75 excluded patients were older patients (53 (i.q.r. 35–63) *versus* 41 (i.q.r. 31–53) years, *P* < 0.001) with comparable morbidity (1.7 per cent permanent RLN palsy and 3.4 per cent permanent hypocalcaemia), comparable disease stage distribution (96.5 per cent TNM stage 1 and 2), but a lower number of harvested LNs (18 (i.q.r. 15–26) *versus* 28 (i.q.r. 20–40), *P <* 0.001; data not shown). Baseline characteristics are shown in *Table 1*. The overall median age at surgery was 41 (i.q.r. 31–53) years, the overall female-to-male ratio was 2.2, and the median BMI was 24 (i.q.r. 21–27) kg/m^2^. No significant differences in age, sex, BMI, or co-morbidity were seen between the cured and the disease event group.

**Table 1:** Patient characteristics

	Cured	Disease event	*P* *	Total cohort
Number of patients, *n* (%)	280 (60.6)	182 (39.4)		462 (100.0)
**Age at surgery, median (i.q.r.), years**	42 (32–54)	39 (29–50)	0.201	41 (31–53)
14–34	98 (35.0)	67 (36.8)	0.364	165 (35.7)
35–46	77 (27.5)	58 (31.9)		135 (29.2)
47–87	105 (37.5)	57 (31.3)		162 (35.1)
**Sex, *n* (%)**				
Female	201 (71.8)	115 (63.2)	0.052	316 (68.4)
Male	79 (28.2)	67 (36.8)		146 (31.6)
**BMI, median (i.q.r.), kg/m²**	24 (21–27)	23 (21–27)	0.478	24 (21–27)
17–22	102 (36.4)	72 (39.6)	0.790	174 (37.7)
23–25	75 (26.8)	47 (25.8)		122 (26.4)
26–45	103 (36.8)	63 (34.6)		166 (35.9)
**Medication, *n* (%)**				
Vitamin K antagonists	5 (1.8)	3 (1.6)	0.912	8 (1.7)
Aspirin	12 (4.3)	10 (5.5)	0.551	22 (4.8)
Steroids	0 (0.0)	2 (1.1)		2 (0.4)
Family history of thyroid cancer, *n* (%)	10 (3.6)	11 (6.0)	0.269	21 (4.5)
**Co-morbidity, *n* (%)**				
Hyperthyroidism, *n* (%)	6 (2.1)	9 (4.9)	0.097	15 (3.2)
Preoperative TSH, median (i.q.r.), mIU/l	1.71 (1.11–2.45)	1.73 (1.13–2.43)		1.73 (1.11–2.44)
Arterial hypertension, *n* (%)	39 (13.9)	24 (13.2)	0.820	63 (13.6)
Hypercholesterolaemia, *n* (%)	31 (11.1)	12 (6.6)	0.105	43 (9.3)
Diabetes, *n* (%)	9 (3.2)	5 (2.7)	0.775	14 (3.0)
Obstructive sleep apnoea syndrome, *n* (%)	7 (2.5)	4 (2.2)	0.835	11 (2.4)

^*^Cured *versus* disease event. LN, lymph nodes; TSH, thyroid-stimulating hormone.

### Postoperative outcome and pathology

Postoperative 30-day and long-term morbidity (after at least one year of follow-up) is shown in *Table 2*. Median follow-up time since surgery was 93 (i.q.r. 50–150) months. Five patients (1.1 per cent) required a re-intervention for bleeding. Thirteen patients (1.4 per cent) developed a permanent, unilateral RLN palsy, and 30 patients (6.5 per cent) suffered from permanent hypocalcaemia. No significant differences were seen in major and minor complications between the cured and the disease event group. Two (0.4 per cent) cancer-related deaths and two (0.4 per cent) non-cancer-related deaths were registered. Both cancer-related deaths were patients who developed distant metastases and de-differentiation of the initial tumour.

**Table 2:** Postoperative morbidity

	Cured	Disease event	*P* *	Total cohort
Number of patients, *n* (%)	280 (60.6)	182 (39.4)		462 (100.0)
**Major complications, *n* (%)**	31 (11.1)	17 (9.3)	0.906†	48 (10.4)
Permanent RLN Injury‡	7 (1.3‡)	6 (1.6‡)	0.613	13 (1.4‡)
Re-intervention for bleeding	4 (1.4)	1 (0.5)	0.372	5 (1.1)
Permanent hypocalcaemia	20 (7.1)	10 (5.5)	0.482	30 (6.5)
**Minor complications, *n* (%)**	78 (27.9)	41 (22.5)		119 (25.8)
Temporary RLN injury‡	11 (2.0‡)	9 (2.5‡)	0.560	20 (2.2‡)
Wound abscess	1 (0.4)	0 (0.0)		1 (0.2)
Temporary hypocalcaemia	66 (23.6)	32 (17.6)	0.124	98 (21.2)
Distant metastasis, *n* (%)	0 (0.0)	17 (9.3)		17 (3.7)
**Mortality (all), *n* (%)**	1 (0.4)	3 (1.6)	0.143	4 (0.9)
Mortality (cancer-related), *n* (%)	0 (0.0)	2 (1.1)		2 (0.4)
Mortality (other), *n* (%)	1 (0.4)	1 (0.5)		2 (0.4)
Time to disease event, median (i.q.r.), months	N/A	7 (3–10)		N/A
Total follow-up time, median (i.q.r), months	105 (55–154)	75 (45–137)		93 (50–149)

^*^Cured *versus* disease event. ‡Calculated on nerves-at-risk. †Major and minor morbidity in cured *versus* disease event. N/A, not applicable; RLN: recurrent laryngeal nerve.

The results of the final pathology reports are presented in *Table 3*. The median weight of the thyroid was 19 (14–27) g. Macrocarcinomas were found in 349 (75.5 per cent) cases. The median maximum tumour size was 15 (11–24) mm with a median number of 3 (1–6) tumour foci. Multifocality was present in 309 patients (66.9 per cent) and bilateral thyroid disease in 199 patients (43.1 per cent). Thyroid capsular and vascular invasion was found in 317 (68.6 per cent) and 131 (28.4 per cent) patients, respectively. Most patients were categorized as TNM stage 1 (*N* = 335, 72.5 per cent).

**Table 3:** Pathology results

	Cured	Disease event	*P* *	Total cohort
Number of patients, *n* (%)	280 (60.6)	182 (39.4)		462 (100.0)
Macrocarcinoma, *n* (%)	204 (72.9)	145 (79.7)	0.096	349 (75.5)
**Maximum tumour size, median (i.q.r.), mm**	15 (10–22)	19 (12–28)	0.004	15 (11–24)
<11 mm, *n* (%)	76 (27.1)	38 (20.9)	0.007	114 (24.7)
11–20, *n* (%)	122 (43.6)	63 (34.6)		185 (40.0)
>20–40, *n* (%)	71 (25.4)	66 (36.3)		137 (29.7)
>40, *n* (%)	11 (3.9)	15 (8.2)		26 (5.6)
Second lesion tumour size, median (i.q.r.), mm	5 (2–7)	3 (2–8)	0.401	4 (2–7)
**Number of tumour foci, median (i.q.r.)**	2 (1–5)	3 (1–11)	<0.001	3 (1–6)
1, *n* (%)	108 (38.6)	47 (25.8)	0.004	155 (33.5)
2–4, *n* (%)	97 (34.6)	63 (34.6)		160 (34.6)
>4, *n* (%)	75 (26.8)	72 (39.6)		147 (31.8)
Multifocality, *n* (%)	173 (61.8)	136 (74.7)	<0.001	309 (66.9)
Bilateral malignant disease, *n* (%)	108 (38.6)	91 (50.0)	0.003	199 (43.1)
Thyroid capsular invasion, *n* (%)	170 (60.7)	147 (80.8)	<0.001	317 (68.6)
Thyroid vascular invasion, *n* (%)	67 (23.9)	64 (35.2)	0.003	131 (28.4)
**Weight of the thyroid, median (i.q.r.), g**	19 (13–25)	20 (15–29)	0.005	19 (14–27)
1–15, *n* (%)	109 (38.9)	46 (25.3)	0.005	155 (33.5)
16–23, *n* (%)	90 (32.1)	62 (34.1)		152 (32.9)
24–478, *n* (%)	81 (28.9)	74 (40.7)		155 (33.5)
**pTNM, *n* (%)**				
T0	1 (0.4)	0 (0.0)	0.004	1 (0.2)
T1a	77 (27.5)	38 (20.9)		115 (24.9)
T1b	115 (41.1)	58 (31.9)		173 (37.4)
T2	66 (23.6)	57 (31.3)		123 (26.6)
T3a	3 (1.1)	5 (2.7)		8 (1.7)
T3b	11 (3.9)	10 (5.5)		21 (4.5)
T4	7 (2.5)	14 (7.7)		21 (4.5)
**Postoperative LN stage, *n* (%)**				
N1a	48 (17.1)	13 (7.1)	0.002	61 (13.2)
N1b	232 (82.9)	169 (92.9)		401 (86.8)
**TNM stage, *n* (%)**				
I	207 (73.9)	128 (70.3)	0.221	335 (72.5)
II	67 (23.9)	44 (24.2)		111 (24.0)
III	3 (1.1)	7 (3.8)		10 (2.2)
IV	3 (1.1)	3 (1.6)		6 (1.3)

^*^Cured *versus* disease event. LN, lymph nodes.

### Risk of recurrence and predictive factors

After a median follow-up of 93 (50–149) months, a total of 182 (39.4 per cent) patients had a disease event. Patients with a disease event presented with significantly larger maximum tumour sizes (19 (i.q.r. 12–28) *versus* 15 (i.q.r. 10–22) mm, *P =* 0.004), more tumour foci (3 (i.q.r. 1–11) *versus* 2 (i.q.r. 1–5), *P* < 0.001), heavier thyroids (20 (i.q.r. 15–29) *versus* 19 (i.q.r. 13–25) g, *P* = 0.005), and more N1b stage disease (17.1 per cent *versus* 7.1 per cent, *P =* 0.002). Seventeen (3.7 per cent) patients developed distant metastasis (*Table 2*).

LN characteristics are shown in *Table 4*. The median number of harvested LNs was 28 (20–40), with a median number of positive LNs of 7 (4–12). LN capsular rupture was seen in 260 patients (56.3 per cent). Median maximum LN size was 15 (9–23) mm. The median central, lateral, and total LNR was 0.50 (0.22–0.75), 0.15 (0.07–0.29), and 0.26 (0.14–0.41), respectively. Patients with a disease event had significantly more harvested LNs (*P =* 0.016); and more positive LNs, positive central and lateral LNs, larger maximum LN sizes, and higher central, lateral, and total LNRs (all *P* < 0.001).

**Table 4:** Lymph node characteristics

	Cured	Disease event	*P* *	Total cohort
Number of patients, *n* (%)	280 (60.6)	182 (39.4)		462 (100.0)
**Number of harvested LN, median (i.q.r.)**	28 (20–37)	31 (21–46)	0.051	28 (20–40)
1–22, *n* (%)	98 (35.0)	55 (30.2)	0.016	153 (33.1)
23–35, *n* (%)	101 (36.1)	51 (28.0)		152 (32.9)
36–94, *n* (%)	81 (28.9)	76 (41.8)		157 (34.0)
<6 LN harvested, *n* (%)	0 (0.0)	1 (0.5)		1 (0.2)
6–9 LN harvested, *n* (%)	4 (1.4)	2 (1.1)	0.811	6 (1.3)
Number of positive LN, median (i.q.r.)	6 (3–9)	10 (6–17)	<0.001	7 (4–12)
Capsular rupture LN, *n* (%)	129 (46.1)	131 (72.0)	<0.001	260 (56.3)
Positive central LN, *n* (%)	236 (84.3)	167 (91.8)	0.019	403 (87.2)
Positive lateral LN, *n* (%)	226 (80.7)	166 (91.2)	0.002	392 (84.8)
Central LN, median (i.q.r.)	7 (4–11)	7 (5–12)	0.150	7 (4–11)
<3 LN harvested, *n* (%)	30 (10.7)	19 (10.4)	0.925	49 (10.6)
Number of positive central LN, median (i.q.r.)	2 (1–5)	4 (2–8)	<0.001	3 (1–6)
Lateral LN, median (i.q.r.)	14 (10–20)	16 (9–24)	0.103	15 (10–21)
<6 LN harvested, *n* (%)	17 (6.1)	12 (6.6)	0.821	29 (6.3)
Number of positive lateral LN, median (i.q.r.)	2 (1–4)	4 (2–7)	<0.001	2 (1–5)
Positive contralateral central LN, *n* (%)	41 (14.6)	55 (30.2)	<0.001	96 (20.8)
Positive contralateral lateral LN, *n* (%)	30 (10.7)	39 (21.4)	<0.001	69 (14.9)
**Maximum LN size, median (i.q.r.), mm†**	12 (8–20)	19 (12–25)	<0.001	15 (9–23)
1–10, *n* (%)	60 (40.0)	27 (20.5)	<0.001	87 (30.9)
11–20, *n* (%)	55 (36.7)	53 (40.2)		108 (38.3)
21–80, *n* (%)	35 (23.3)	52 (39.4)		87 (30.9)
**Central LN ratio, median (i.q.r.)**	0.38 (0.17–0.65)	0.67 (0.40–0.87)	<0.001	0.50 (0.22–0.75)
0.00–0.33, *n* (%)	131 (46.8)	39 (21.4)	<0.001	170 (36.8)
0.34–0.69, *n* (%)	95 (33.9)	59 (32.4)		154 (33.3)
0.70–1.00, *n* (%)	54 (19.3)	84 (46.2)		138 (29.9)
**Lateral LN ratio, median (i.q.r.)**	0.11 (0.05–0.22)	0.23 (0.11–0.38)	<0.001	0.15 (0.07–0.29)
0.00–0.10, *n* (%)	127 (45.4)	43 (23.6)	<0.001	170 (36.8)
0.11–0.25, *n* (%)	99 (35.4)	59 (32.4)		158 (34.2)
0.26–1.00, *n* (%)	54 (19.3)	80 (44.0)		134 (29.0)
**Total LN ratio, median (i.q.r.)**	0.20 (0.11–0.32)	0.38 (0.23–0.50)	<0.001	0.26 (0.14–0.41)
0.00–0.20, *n* (%)	141 (50.4)	35 (19.2)	<0.001	176 (38.1)
0.21–0.40, *n* (%)	97 (34.6)	65 (35.7)		162 (35.1)
0.41–1.00, *n* (%)	42 (15.0)	82 (45.1)		124 (26.8)

^*^Cured *versus* disease event. †Missing data in 180 (39.0 per cent) patients. LN, lymph nodes.

True recurrent disease was found in 9 (1.9 per cent) patients, biochemical persistence in 48 (10.4 per cent) patients, and structural persistence in 99 (21.4 per cent) patients. No data about type of disease event were available for 26 (5.6 per cent) patients. Structural persistence was mainly seen in the lateral compartment (*n* = 73, 73.3 per cent). Only three (6.2 per cent) patients with biochemical persistence underwent RAI in the year following their surgery, while the others were kept under surveillance. Most patients with structural persistence underwent redo surgery (*n* = 71, 71.7 per cent). Median time to true recurrence was 31 (21–51) months.

**Fig. 2:**
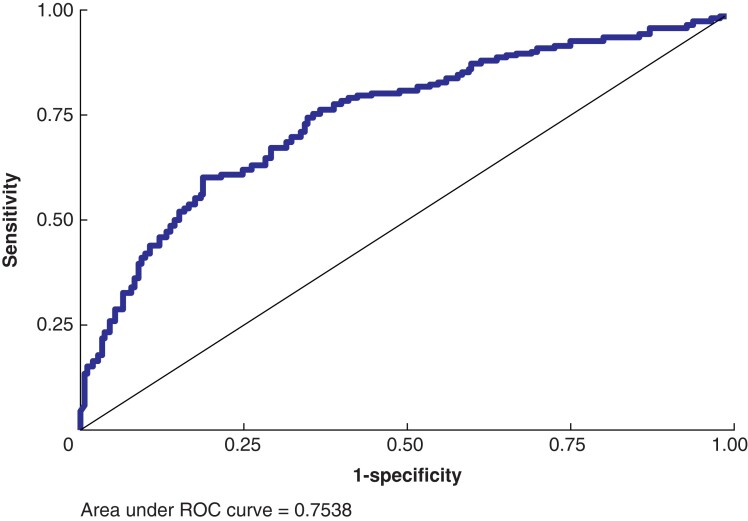
ROC curve of lymph node characteristics

Factors associated with a disease event after surgery in the univariable logistic regression analysis were tumour size >20 mm; >4 tumour foci; multifocality; bilateral malignant disease; thyroid capsular and vascular invasion; weight of the thyroid >23 g; >35 harvested LNs; >4 positive LNs; presence of LN capsular rupture; maximum LN size >10 mm; and central, lateral, and total LNRs above 0.33, 0.10, and 0.20, respectively (*Table 5*, *Table S1*). On multivariable logistic regression, factors associated with a disease event were TNM stage 3 (OR 6.77 (1.25–36.73)), number of harvested LNs >35 (OR 2.35 (1.10–5.02)), presence of LN capsular rupture (OR 1.67 (1.02–2.67)), and total LNR >0.20–0.40 (OR 2.41 (1.09–5.34)) and >0.40 (OR 5.10 (1.65–15.78)). A total LNR of 0.20 predicts a disease event with a sensitivity of 80.8 per cent and a specificity of 50.4 per cent. The presence of LN capsular rupture predicts a disease event with a sensitivity of 72.0 per cent and a specificity of 53.9 per cent. The receiver operating characteristic (ROC) curve for all three factors combined (total LNR, presence of LN capsular rupture, and number of harvested LNs) is shown in *Fig. 2* (area under the curve 0.754, 95 per cent c.i. 0.711–0.797).

**Table 5:** Statistical analyses

	Univariable logistic regression for risk of disease event		Multivariable logistic regression for risk of disease event	
	OR	95% c.i.	OR	95% c.i.
**Sex**				
Female	1		1	
Male	1.48	1.00–2.21	0.93	0.55–1.57
**Age, years**				
14–34	1		1	
35–46	1.10	0.69–1.75	1.16	0.66–2.03
47–87	0.79	0.51–1.24	0.69	0.34–1.38
**Maximum tumour size, mm**				
< 11	1		1	
11–20	1.03	0.63–1.69	0.64	0.35–1.18
>20–40	**1.86**	**1.11–3.11**	0.90	0.46–1.76
>40	**2.73**	**1.14–6.51**	0.94	0.29–3.00
**Number of tumour foci**				
1	1		1	
2–4	1.49	0.94–2.38	1.23	0.01–177.18
>4	**2.21**	**1.38–3.53**	1.00	0.01–144.84
**Bilateral malignant disease**				
No	1		1	
Yes	**1.59**	**1.09–2.32**	1.12	0.62–2.04
**Thyroid capsular invasion**				
No	1		1	
Yes	**2.72**	**1.75–4.22**	1.66	0.97–2.87
**Thyroid vascular invasion**				
No	1		1	
Yes	**1.72**	**1.14–2.60**	0.82	0.48–1.39
**Weight of the thyroid, g**				
1–15	1		1	
16–23	**1.61**	**1.00–2.58**	1.38	0.78–2.46
24–478	**2.19**	**1.38–3.50**	1.46	0.77–2.79
**TNM stage**				
I	1		1	
II	1.06	0.68–1.65	1.31	0.66–2.64
III	3.77	0.96–14.85	**6.77**	**1.25–36.73**
IV	1.62	0.32–8.13	1.43	0.22–9.20
**Number of harvested LN**				
1–22	1		1	
23–35	0.90	0.56–1.44	1.18	0.63–2.21
36–94	**1.67**	**1.06–2.64**	**2.35**	**1.10–5.02**
**Number of positive LN**				
0–4	1		1	
5–10	**2.06**	**1.26–3.38**	0.69	0.34–1.39
11–58	**5.96**	**3.55–10.01**	0.63	0.22–1.80
**Capsular rupture LN**				
No	1		1	
Yes	**3.01**	**2.02–4.48**	**1.67**	**1.02–2.76**
**Maximum LN size, mm**				
1–10	1		1	
11–20	**2.22**	**1.23–4.03**	1.62	0.80–3.25
21–80	**3.43**	**1.83–6.43**	1.56	0.73–3.31
Missing	0.91	0.52–1.59	0.69	0.35–1.36
**Central LN ratio**				
0.00–0.33	1		1	
0.34–0.69	**2.09**	**1.29–3.38**	1.15	0.60–2.19
0.70–1.00	**5.22**	**3.19–8.57**	1.84	0.86–3.93
**Lateral LN ratio**				
0.00–0.10	1		1	
0.11–0.25	**1.76**	**1.10–2.82**	1.02	0.54–1.90
0.26–1.00	**4.38**	**2.68–7.13**	1.34	0.58–3.06
**Total LN ratio**				
0.00–0.20	1		1	
0.21–0.40	**2.70**	**1.66–4.39**	**2.41**	**1.09–5.34**
0.41–1.00	**7.87**	**4.65–13.29**	**5.10**	**1.65–15.78**

Bold values are significant results, as the 1 value does not lie within the confidence intervals. LN, lymph node.

Due to a low number of events (nine patients), univariable Cox regression analysis assessing factors associated with true recurrence could not identify significant associations (apart from maximum LN size, with wide 95 per cent c.i.s; *Table 5*).

Overall mortality of the total cohort was low (*N* = 5, 1.1 per cent) with a disease-specific survival (DSS) of 99.0 per cent in the group of patients with already at least 60 months follow-up after surgery. The DSS at 10 years was 99.2 per cent (119/120) for TNM stage I, 97.6 per cent (41/42) for TNM stage II, 100 per cent (5/5) for TNM stage III, and 66.6 per cent (2/3) for TNM stage IV.

## Discussion

This retrospective study of a prospective surgical cohort analysed the risk of disease events after total thyroidectomy with therapeutic central and lateral neck dissection followed by radioactive iodine for classic PTC with preoperative TNM N1 nodal disease. Multivariable logistic regression analysis of 462 consecutive patients showed that the number of harvested LNs >35, presence of LN capsular rupture, and total LNR >0.20–0.40 and >0.40 were statistically significant factors for disease events. TNM stage 3 was statistically significant as well, bearing in mind the wide confidence interval as only 10 patients were analysed (and only 6 in TNM stage 4). No significant differences in morbidity between both study groups were found. These factors should be incorporated in future prediction models and clinical decision making.

To the authors' knowledge, this is the largest study evaluating a homogenous cohort of clinically node-positive classic PTC. The inclusion of all consecutive patients reduces the risk of selection bias. The extensive experience of the surgical department with cancer and non-cancer-related thyroid surgery, the standardized work-up, surgical approach and treatment, and the completeness of the data set strengthen the analysis and results.

Despite the growing evidence that metastatic LNR is a valuable predictor for the prognosis of PTC, how this can be incorporated into current TNM and ATA classification and grading systems has not yet been clearly defined. A systematic review found nine retrospective studies showing that regional LNR is an independent predictor for loco-regional recurrence in pathologically staged N1 patients with PTC^12^. In addition, a recent study confirmed that LNR is an independent and better predictor of recurrence than the AJCC/UICC TNM N stage^14^. They found that an LNR of 0.15 was related to tumour recurrence with a sensitivity of 69 per cent and a specificity of 59 per cent. Our study confirms the importance of the total LNR compared to maximum LN size and number of positive LN and validates these previous studies in a larger, more homogenous surgical cohort. LN capsular rupture (or ‘extranodal growth’) was previously identified as a risk factor for disease recurrence^15^. In the current series, presence of capsular rupture predicted a disease event with a sensitivity of 72 per cent, which strengthens the importance of this variable as a risk factor for disease recurrence and persistence. The relationship between the number of harvested LN and a disease event might be surprising, but probably reflects a more aggressive surgical resection in patients with multiple preoperatively known LN metastasis. The clinical significance of extensive lateral neck dissection in limited lateral disease remains controversial and should always be viewed in the light of possible morbidity and little to no survival benefit.

The disease event of 39 per cent appears high but is comparable to other series when including preoperative N1 disease. A Saudi Arabian study reported a recurrence of 27 per cent, excluding persistent disease^14^. Analysis of a Korean population showed a recurrence rate of 19 per cent, excluding biochemical recurrence^16^. A recent study, analysing 4292 consecutive patients with all stages of differentiated thyroid cancer, separated persistent and recurrent disease^17^. A disease event as defined in our series was reported in 34 per cent of patients. In patients with pathological proven cervical LN metastases, the median risk of loco-regional LN recurrence for patients who are initially clinically N-positive (cN1) was 22 per cent (range 10 –42 per cent)^18^. Moreover, the term ‘recurrence’ is used inconsistently in scientific literature. Many studies fail to mention the time to recurrence and early recurrences are likely to represent persistence^13^. Moreover, the relevance of biochemical persistence is not clear. The difference between persistent and recurrent disease is clinical, with a disease event identifiable from the start (structurally or biochemically persistent) or a disease event that becomes clinically apparent after a certain time (recurrent disease). Persistence is the consequence of more aggressive and advanced tumours, and because of incomplete initial treatment.

Most patients with structural persistence underwent redo surgery (71.7 per cent). A recent analysis of 69 patients requiring reoperation confirmed that most procedures were performed managing persistent disease (96.8 per cent), with over half of the patients requiring a reoperation within the first two years. This shows that improvements in the preoperative assessment and adequacy of initial surgery are to be made for better care of patients with thyroid cancer^13^.

These results must be interpreted with caution. As stated in the methods section, biologically aggressive variants of PTC (hobnail, tall cell, columnar, and solid variants) were excluded from the analysis. The Ki-67 proliferation index might also reflect the malignant behaviour of PTC, but could not be evaluated in this series^8^.

Future research and efforts are necessary and should be aimed at reducing the frequency of persistent and recurrent disease by better characterizing the cancer genotype, by improving preoperative assessment and the adequacy of initial surgery, and by aiming for a personalized post-initial treatment follow-up and management. Data collection at national and international levels is required to be able to construct and refine predictive models that include a clinically useful LNR in order to prognosticate which patients require more intense follow-up.

## Supplementary Material

zrad124_Supplementary_Data

## Data Availability

The (raw) data that support the findings of this study are available on reasonable request from the senior author (F.M.).
