# Supplementary material for: Prognostic impact of lymph node characteristics after therapeutic neck dissection for classic N1 papillary thyroid cancer
Source: BJS Open. 2023 Nov 28;7(6):zrad124. doi: 10.1093/bjsopen/zrad124 (PMC10684262; doi:10.1093/bjsopen/zrad124)
Supplement: zrad124_Supplementary_Data [file zrad124_supplementary_data.zip › zrad124_Supplementary_Data.docx]

**Prognostic Impact of Lymph Node Characteristics after Therapeutic Neck Dissection for Classic N1 Papillary Thyroid Cancer**

Klaas Van Den Heede, MD^1,2^, Nele Brusselaers, MD, PhD^3,4^, Esmee Breddels^4^, Sébastien Gaujoux, MD, PhD^1,5^, Camille Buffet MD, PhD^5,6^ , Fabrice Menegaux MD, PhD^1,5^, Nathalie Chereau MD^1,5^

1/ Department of General and Endocrine Surgery, Pitié Salpêtrière Hospital, APHP, Sorbonne University, 47-83 Boulevard de l'Hôpital, Paris, France

2/ Department of General and Endocrine Surgery, Onze-Lieve-Vrouw (OLV) Ziekenhuis Aalst, Aalst, Belgium

3/ Center for Translational Microbiome Research Department of Microbiology, Tumor and Cell Biology, Karolinska Institute, Stockholm, Sweden

4/ Global Health Institute, University of Antwerp, Wilrijk, Belgium

5/ Groupe de Recherche Clinique n°16 Thyroid Tumors, Sorbonne University, Paris, France

6/ Thyroid and Endocrine Tumor Unit, Pitié Salpêtrière Hospital, APHP, Sorbonne University, Paris, France.

**Corresponding author.** Klaas Van Den Heede, MD, Pitié Salpêtrière Hospital, APHP, Sorbonne University - Department of General and Endocrine Surgery **-** 47-83 Boulevard de l'Hôpital, Paris – ORCID 0000-0003-3642-9514

**Supplementary Materials - Index**

| **Supplementary Methods** |  |
| --- | --- |
| Detail | *pag. X* |
| Detail | *pag. Y* |
| **Supplementary Results** |  |
| Full Statistical Analyses | *pag. 6* |
| Detail | *pag. Y* |
| **Supplementary Appendixes** |  |
| Detail | *pag. X* |
| Detail | *pag. Y* |
| **Supplementary Figures and Tables** |  |
| Detail | *pag. X* |
| Detail | *pag. Y* |
| **References** | *pag. Z* |
|  |  |

**Supplementary Methods**

**Supplementary Results**

| **Supplementary Table 2 Full Statistical Analyses** |  |  |  |  |  |  |
| --- | --- | --- | --- | --- | --- | --- |
|  | Univariable Logistic Regression for Risk of Disease Event | | Multivariable Logistic Regression for Risk of Disease Event | | Univariable Cox Regression for Risk of True Recurrence | |
|  | OR | 95% CI | OR | 95% CI | HR | 95% CI |
| Sex |  |  |  |  |  |  |
| ° Female | 1 |  | 1 |  | 1 |  |
| ° Male | 1.48 | 1.00-2.21 | 0.93 | 0.55-1.57 | 0.73 | 0.15-3.52 |
| Age, years |  |  |  |  |  |  |
| ° 14-34 | 1 |  | 1 |  | 1 |  |
| ° 35-46 | 1.10 | 0.69-1.75 | 1.16 | 0.66-2.03 | 2.00 | 0.48-8.35 |
| ° 47-87 | 0.79 | 0.51-1.24 | 0.69 | 0.34-1.38 | 0.31 | 0.03-2.95 |
| BMI, kg/m² |  |  |  |  |  |  |
| ° 17-22 | 1 |  |  |  | 1 |  |
| ° 23-25 | 0.89 | 0.55-1.43 |  |  | 4.09 | 0.43-39.33 |
| ° 26-45 | 0.87 | 0.56-1.34 |  |  | 5.01 | 0.59-42.92 |
| Pathology |  |  |  |  |  |  |
| ° Macrocarcinoma | 1 |  |  |  | 1 |  |
| ° Microcarcinoma | 0.68 | 0.44-1.07 |  |  | 0.34 | 0.04-2.73 |
| Maximum Tumor Size, mm |  |  |  |  |  |  |
| ° <11mm | 1 |  | 1 |  | 1 |  |
| ° 11-20 | 1.03 | 0.63-1.69 | 0.64 | 0.35-1.18 | 1.85 | 0.19-17.81 |
| ° >20-40 | 1.86 | 1.11-3.11 | 0.90 | 0.46-1.76 | 5.09 | 0.60-43.61 |
| ° >40 | 2.73 | 1.14-6.51 | 0.94 | 0.29-3.00 | <0.01 | N/A |
| Number of Tumor Foci |  |  |  |  |  |  |
| ° 1 | 1 |  | 1 |  | 1 |  |
| ° 2-4 | 1.49 | 0.94-2.38 | 1.23 | 0.01-177.18 | 2.17 | 0.40-11.84 |
| ° >4 | 2.21 | 1.38-3.53 | 1.00 | 0.01-144.84 | 2.04 | 0.34-12.23 |
| Multifocality |  |  |  |  |  |  |
| ° No | 1 |  | 1 |  | 1 |  |
| ° Yes | 1.83 | 1.21-2.76 | 1.13 | 0.01-169.91 | 2.07 | 0.43-9.98 |
| Bilateral Malignant Disease |  |  |  |  |  |  |
| ° No | 1 |  | 1 |  | 1 |  |
| ° Yes | 1.59 | 1.09-2.32 | 1.12 | 0.62-2.04 | 0.82 | 0.20-3.27 |
| Thyroid Capsular Invasion |  |  |  |  |  |  |
| ° No | 1 |  | 1 |  | 1 |  |
| ° Yes | 2.72 | 1.75-4.22 | 1.66 | 0.97-2.87 | 2.04 | 0.42-9.84 |
| Thyroid Vascular Inviasion |  |  |  |  |  |  |
| ° No | 1 |  | 1 |  | 1 |  |
| ° Yes | 1.72 | 1.14-2.60 | 0.82 | 0.48-1.39 | 1.12 | 0.23-5.43 |
| Weight of the Thyroid, grams |  |  |  |  |  |  |
| ° 1-15 | 1 |  | 1 |  | 1 |  |
| ° 16-23 | 1.61 | 1.00-2.58 | 1.38 | 0.78-2.46 | 4.79 | 0.53-42.83 |
| ° 24-478 | 2.19 | 1.38-3.50 | 1.46 | 0.77-2.79 | 5.55 | 0.62-49.63 |
| TNM Stage |  |  |  |  |  |  |
| ° I | 1 |  | 1 |  | 1 |  |
| ° II | 1.06 | 0.68-1.65 | 1.31 | 0.66-2.64 | 1.47 | 0.37-5.90 |
| ° III | 3.77 | 0.96-14.85 | 6.77 | 1.25-36.73 | <0.01 | N/A |
| ° IV | 1.62 | 0.32-8.13 | 1.43 | 0.22-9.20 | <0.01 | N/A |
| Number of Harvested LN |  |  |  |  |  |  |
| ° 1-22 | 1 |  | 1 |  | 1 |  |
| ° 23-35 | 0.90 | 0.56-1.44 | 1.18 | 0.63-2.21 | 0.46 | 0.08-2.51 |
| ° 36-94 | 1.67 | 1.06-2.64 | 2.35 | 1.10-5.02 | 0.77 | 0.17-3.47 |
| Number of Positive LN |  |  |  |  |  |  |
| ° 0-4 | 1 |  | 1 |  | 1 |  |
| ° 5-10 | 2.06 | 1.26-3.38 | 0.69 | 0.34-1.39 | 1.07 | 0.22-5.31 |
| ° 11-58 | 5.96 | 3.55-10.01 | 0.63 | 0.22-1.80 | 2.40 | 0.48-11.90 |
| Capsular Rupture LN |  |  |  |  |  |  |
| ° No | 1 |  | 1 |  | 1 |  |
| ° Yes | 3.01 | 2.02-4.48 | 1.67 | 1.02-2.76 | 2.19 | 0.55-8.78 |
| Maximum LN Size, mm |  |  |  |  |  |  |
| ° 1-10 | 1 |  | 1 |  | 1 |  |
| ° 11-20 | 2.22 | 1.23-4.03 | 1.62 | 0.80-3.25 | <0.01 | 0.00-0.00 |
| ° 21-80 | 3.43 | 1.83-6.43 | 1.56 | 0.73-3.31 | <0.01 | 0.00-0.00 |
| ° Missing | 0.91 | 0.52-1.59 | 0.69 | 0.35-1.36 | <0.01 | N/A |
| Central LN Ratio |  |  |  |  |  |  |
| ° 0.00-0.33 | 1 |  | 1 |  | 1 |  |
| ° 0.34-0.69 | 2.09 | 1.29-3.38 | 1.15 | 0.60-2.19 | 7.06 | 0.82-60.41 |
| ° 0.70-1.00 | 5.22 | 3.19-8.57 | 1.84 | 0.86-3.93 | 7.55 | 0.79-72.61 |
| Lateral LN Ratio |  |  |  |  |  |  |
| ° 0.00-0.10 | 1 |  | 1 |  | 1 |  |
| ° 0.11-0.25 | 1.76 | 1.10-2.82 | 1.02 | 0.54-1.90 | 2.96 | 0.54-16.19 |
| ° 0.26-1.00 | 4.38 | 2.68-7.13 | 1.34 | 0.58-3.06 | 3.77 | 0.62-22.60 |
| Total LN Ratio |  |  |  |  |  |  |
| ° 0.00-0.20 | 1 |  | 1 |  | 1 |  |
| ° 0.21-0.40 | 2.70 | 1.66-4.39 | 2.41 | 1.09-5.34 | 3.22 | 0.59-17.57 |
| ° 0.41-1.00 | 7.87 | 4.65-13.29 | 5.10 | 1.65-15.78 | 5.59 | 0.93-33.50 |
|  |  |  |  |  |  |  |
| *CI: confidence interval - LN: lymph node - OR: odds ratio* | | |  |  |  |  |

**Supplementary Appendixes**

**Supplementary Figures and Tables**

**References**
