# Supplementary material for: Prognostic impact of lymph node characteristics after therapeutic neck dissection for classic N1 papillary thyroid cancer
Source: BJS Open. 2023 Nov 28;7(6):zrad124. doi: 10.1093/bjsopen/zrad124 (PMC10684262; doi:10.1093/bjsopen/zrad124)
Supplement: zrad124_Supplementary_Data [file zrad124_supplementary_data.zip › Supplementary table 1.docx]

| **Supplementary table 1 Patient characteristics** |  |  |  |  |
| --- | --- | --- | --- | --- |
|  | Cured | Disease Event | p-value | Total Cohort |
| Number of Patients, No. (%) | 280 (60.6) | 182 (39.4) |  | 462 (100.0) |
|  |  |  |  |  |
| Age at Surgery, median (IQR), years | 42 (32-54) | 39 (29-50) | .201 | 41 (31-53) |
| ° 14-34 | 98 (35.0) | 67 (36.8) | .364 | 165 (35.7) |
| ° 35-46 | 77 (27.5) | 58 (31.9) |  | 135 (29.2) |
| ° 47-87 | 105 (37.5) | 57 (31.3) |  | 162 (35.1) |
| Sex, No. (%) |  |  |  |  |
| ° Female | 201 (71.8) | 115 (63.2) | .052 | 316 (68.4) |
| ° Male | 79 (28.2) | 67 (36.8) |  | 146 (31.6) |
| Female/Male Ratio | 2.5 | 1.7 |  | 2.2 |
| BMI, median (IQR), kg/m² | 24 (21-27) | 23 (21-27) | .478 | 24 (21-27) |
| ° 17-22 | 102 (36.4) | 72 (39.6) | .790 | 174 (37.7) |
| ° 23-25 | 75 (26.8) | 47 (25.8) |  | 122 (26.4) |
| ° 26-45 | 103 (36.8) | 63 (34.6) |  | 166 (35.9) |
| Medication, No. (%) |  |  |  |  |
| ° Vitamin K Antagonists | 5 (1.8) | 3 (1.6) |  | 8 (1.7) |
| ° Aspirin | 12 (4.3) | 10 (5.5) |  | 22 (4.8) |
| ° Steroids | 0 (0.0) | 2 (1.1) |  | 2 (0.4) |
| Family History of Thyroid Cancer, No. (%) | 10 (3.6) | 11 (6.0) |  | 21 (4.5) |
| COMORBIDITY |  |  |  |  |
| Hyperthyroidism, No. (%) | 6 (2.1) | 9 (4.9) |  | 15 (3.2) |
| ° Preoperative TSH, median (IQR), mIU/L | 1.71 (1.11-2.45) | 1.73 (1.13-2.43) |  | 1.73 (1.11-2.44) |
| Arterial hypertension, No. (%) | 39 (13.9) | 24 (13.2) |  | 63 (13.6) |
| Hypercholesterolemia, No. (%) | 31 (11.1) | 12 (6.6) |  | 43 (9.3) |
| Diabetes, No. (%) | 9 (3.2) | 5 (2.7) |  | 14 (3.0) |
| Obstructive Sleep Apnea Syndrome, No. (%) | 7 (2.5) | 4 (2.2) |  | 11 (2.4) |
|  |  |  |  |  |
| *IQR: interquartile range - LN: lymph nodes - No: number - TSH: thyroid-stimulating hormone* | | | | |
